# Surviving Reactive Chlorine Stress: Responses of Gram-Negative Bacteria to Hypochlorous Acid

**DOI:** 10.3390/microorganisms8081220

**Published:** 2020-08-11

**Authors:** Waleska Stephanie da Cruz Nizer, Vasily Inkovskiy, Joerg Overhage

**Affiliations:** Department of Health Sciences, Carleton University, Ottawa, ON K1S 5B6, Canada; waleskadacruznizer@cmail.carleton.ca (W.S.d.C.N.); VasilyInkovskiy@cmail.carleton.ca (V.I.)

**Keywords:** hypochlorous acid, sodium hypochlorite, stress response, Gram-negative bacteria, pathogens, household bleach, VBNC, antimicrobial resistance, oxidative stress, reactive chlorine species

## Abstract

Sodium hypochlorite (NaOCl) and its active ingredient, hypochlorous acid (HOCl), are the most commonly used chlorine-based disinfectants. HOCl is a fast-acting and potent antimicrobial agent that interacts with several biomolecules, such as sulfur-containing amino acids, lipids, nucleic acids, and membrane components, causing severe cellular damage. It is also produced by the immune system as a first-line of defense against invading pathogens. In this review, we summarize the adaptive responses of Gram-negative bacteria to HOCl-induced stress and highlight the role of chaperone holdases (Hsp33, RidA, Cnox, and polyP) as an immediate response to HOCl stress. We also describe the three identified transcriptional regulators (HypT, RclR, and NemR) that specifically respond to HOCl. Besides the activation of chaperones and transcriptional regulators, the formation of biofilms has been described as an important adaptive response to several stressors, including HOCl. Although the knowledge on the molecular mechanisms involved in HOCl biofilm stimulation is limited, studies have shown that HOCl induces the formation of biofilms by causing conformational changes in membrane properties, overproducing the extracellular polymeric substance (EPS) matrix, and increasing the intracellular concentration of cyclic-di-GMP. In addition, acquisition and expression of antibiotic resistance genes, secretion of virulence factors and induction of the viable but nonculturable (VBNC) state has also been described as an adaptive response to HOCl. In general, the knowledge of how bacteria respond to HOCl stress has increased over time; however, the molecular mechanisms involved in this stress response is still in its infancy. A better understanding of these mechanisms could help understand host-pathogen interactions and target specific genes and molecules to control bacterial spread and colonization.

## 1. Introduction

The emerging rise of antimicrobial resistant Gram-negative bacteria has become the major cause of concern for global public health [1]. In this context, an effective way to control bacterial colonization and spread is the use of disinfectants. For instance, due to the broad spectrum of antimicrobial action, the WHO recommends the use of sodium hypochlorite (NaOCl) to disinfect surfaces to control the spread of microbial infections, mainly in hospital settings [2].

Gram-negative bacteria cause a wide range of serious infections, including pneumonia, urinary tract infections (UTIs), bloodstream, intra-abdominal, and wound infections [1]. Gram-negative pathogens are often more resistant to different classes of antibiotics than Gram-positive bacteria, mainly due to the presence of an impermeable outer membrane, which confers protection to the cells against hostile environmental conditions [3,4]. Furthermore, horizontal gene transfer and the expression of several efflux pumps decrease the susceptibility of these pathogens to the effect of antimicrobial agents [5,6].

In their natural environment, bacteria often form biofilms, which are cellular aggregates attached to surfaces. These structures are embedded in a matrix of a self-produced, extracellular polymeric substance (EPS), consisting of extracellular DNA (eDNA), polysaccharides, and proteins [7,8]. In general, the formation of a biofilm is a complex and fluid process that starts with the attachment of free-swimming cells to a surface. Then, bacterial cells begin multiplying and producing the EPS matrix and form microcolonies, which will subsequently become the mature biofilm. Finally, biofilm disrupts, and single cells or clusters of cells can leave to form new biofilms and spread [9]. Several genes are regulated during this process, including those involved in the production of the extracellular matrix components (e.g., extracellular enzymes, DNA, and polysaccharides), adhesion proteins, translation (e.g., ribosomal protein and translation initiation factor), membrane or secretion proteins (e.g., translocation proteins, porins, and lipoproteins), structural proteins (e.g., OmpC, OmpF, OmpT), and repression of motility factors (e.g., flagellar hook protein FlgE and pilin protein PilA) [10,11].

Biofilms are involved in a variety of severe infections, including cystic fibrosis pneumonia, chronic wound, and medical device-related infections [12]. It is estimated that bacteria within biofilms cause approximately 60–80% of all persistent bacterial infections [9]. The formation of biofilms has also been associated with the pathogenicity of bacteria that cause community-acquired infections. For instance, urinary tract infection (UTI) caused by uropathogenic *Escherichia coli* (UPEC) is considered the most common community-acquired infection [13,14]. Moreover, the colonization of surfaces and industrial equipment by these structures in food industries can cause considerable economic losses [15]. For instance, the foodborne pathogen *E. coli* O157:H7 is commonly isolated in meat processing industries, where this pathogen forms robust biofilms on stainless steel surfaces and becomes the causative agent of meat contamination and foodborne infections [16]. Despite the negative aspects of biofilms, they can also be beneficial [17]. They are crucial in several industrial processes, such as wastewater treatment [18], water decontamination by bioremediation [19], and plant protection and nutrient acquisition [20].

Bacteria within biofilms exhibit distinct phenotypes when compared to planktonic cells. Furthermore, these structures are resistant to several environmental stressors, especially antimicrobial agents and the host immune system. For instance, bacteria within biofilms are 100–1000 times more tolerant to antibiotics than planktonic cells [9,21]. There are several reasons for this increased tolerance, including the low metabolic activity of the cells, in which some cells enter in a stationary growth phase [22]; oxygen limitation, which favors the development of anaerobic microbes [23]; high rates of mutations and transfer of resistant plasmids [24]; the presence of the EPS matrix, which protects the cells against environmental stressors [25], and the secretion of quorum sensing (QS) molecules [26].

The QS system or cell-to-cell communication mechanism is known to play an important role in biofilm formation, antibiotic resistance, and production of virulence factors in several bacterial species [27,28,29]. The QS system regulates cell-to-cell communication by the interaction of QS signal molecules with transcriptional regulators, which induces the expression of target genes. *Pseudomonas aeruginosa*, for example, has three well-characterized QS systems: (*las*, *rhl*, and *Pseudomonas* quinolone signal (PQS)) that are involved in biofilm formation and biofilm architecture by the expression of several molecules, such as elastase, proteases, and polysaccharide [28,29]. Furthermore, the QS system is also involved in the formation of pili, flagella, and types II and III secretion systems, which play an important role in attachment and microcolony formation within biofilms [29,30]. Recently, much effort has been made to control and prevent biofilm colonization in domestic, industrial, and clinical settings.

In their different environments, bacteria are constantly confronted by environmental stressors, such as extremes of pH and temperature, osmotic pressure, antimicrobials, and oxidizing agents, which can irreversibly damage the cells. Among the most widespread oxidizing agents, reactive oxygen species (ROS), such as hydrogen peroxide, and reactive chlorine species (RCS), such as hypochlorous acid, are the most abundant. Of these, NaOCl, which is more commonly known as household bleach, is the most used chlorine-based disinfectant [31]. Its reaction with water produces hypochlorous acid (HOCl) (Equation (1)). In aqueous solution of NaOCl, chlorine exists in the forms of chlorine gas (Cl_2_), hypochlorous acid (HOCl), and hypochlorite ion (^−^OCl) in equilibrium (Equation (2)) [32]. Moreover, HOCl, ^−^OCl, and Cl_2_ are often referred to as free chlorine or free available chlorine (FAC), and their relative proportions depend on the pH of the solution. The higher concentration of HOCl can be found at a pH between 4 and 6. At pH values lower than 4, Cl_2_ becomes the predominant chlorine specie. At higher pH values (between 8.5–10), the concentration of HOCl decreases close to zero, and ^−^OCl becomes the major component of the solution [31,32]. Among these forms of chlorine, HOCl has the greatest germicidal action (approximately 80 times greater than ^−^OCl) and determines the activity of diluted NaOCl solution since it is a neutrally charged and can easily penetrate the lipid bilayer of the membrane [32].

(1) $$\mathrm{NaOCl}+H_{2}O\to \mathrm{HOCl}+\mathrm{NaOH}$$

(2) $${\mathrm{Cl}}_{2}⇌\mathrm{HOCl}⇌{\mathrm{OCl}}^{-}$$

In this article, we review the antibacterial mechanisms of action of HOCl. In addition, some studies that evaluated the effect of NaOCl on bacterial cells were also the focus of this review since HOCl is considered the most active ingredient of NaOCl. We also summarize the adaptive strategies adopted by Gram-negative cells to sense and respond to the cellular damage caused by these RCS. First, we describe the role of chaperone holdases as the immediate response to HOCl-induced oxidative stress. Then, we focus on the genetic adjustments undergone by bacterial cells to adapt to HOCl exposure. Herein, we describe three transcriptional regulators (HypT, RclR, and NemR) that respond to RCS, and more specifically, to HOCl. Finally, we discuss the formation of biofilms, the acquisition of antibiotic resistance, and the secretion of virulence factors as survival strategies against the damage caused by HOCl on bacterial cells.

## 2. Hypochlorous Acid, a Widely Used Disinfectant

NaOCl and its active ingredient HOCl are widely used for sanitation and disinfection purposes in industrial, hospital, and household settings [33]. It is frequently used in water disinfection, for example, in swimming pools and in the water treatment process, in which its concentration must follow guidelines specified by regulatory agencies. For instance, the German Institute for Standardization (DIN 19643) indicates that the concentration of free chlorine in pool water must be kept between 0.3–0.6 mg/L [34].

Given its strong oxidizing properties associated with its low cost, HOCl is frequently used in water and wastewater treatment [31]. In drinking water treatment, it is the most commonly used disinfectant and is usually applied in a two-step process: at the beginning of the treatment as a primary disinfectant to inactivate microbial pathogens, and at the end of the process, in which chlorine is added to maintain a residual concentration throughout the water distribution system [35]. This process allows to control the growth of microbes and might also indicate the malfunction or loss of the integrity of the water treatment system. In the USA, the concentration of free chlorine at the entry of the distribution system must be not smaller than 0.2 mg/L [36]. Furthermore, according to Health Canada (2009), NaOCl free chlorine concentration ranges between 0.4 and 2 mg/L in the drinking water leaving the treatment plant, between 0.4 and 1.2 mg/L at the intermediate points, and between 0.04 and 0.8 mg/L at the far ends of the drinking water distribution system. In general, the level of residual chlorine required to control bacterial regrowth in the drinking water distribution system is 0.2 mg/L [37].

The use of NaOCl as a disinfection agent to clean surfaces and medical equipment has increased during the current pandemic caused by the severe acute respiratory syndrome coronavirus 2 (SARS-CoV-2) [38]. According to Health Canada, the number of exposures to bleach, hand sanitizers, disinfectants, and other cleaning products has increased by 58% in March 2020 when compared to March 2019 [39]. SARS-CoV-2 can persist on inanimate materials for hours to days but can be inactivated by a 0.1% solution of NaOCl [38]. The effectiveness of NaOCl to disinfect several viruses, including Ebola virus, Norwalk virus, murine norovirus, and human immunodeficiency virus (HIV) has also been reported [40]. Enveloped viruses, such as SARS-CoV-2, are inactivated by NaOCl due to its interaction with the viral outer lipid envelope [41]. Besides its use to clean surfaces, NaOCl has also been used as a spray to clean outdoor spaces, such as streets, and in disinfectant chambers; however, both uses are not recommended since NaOCl can react with organic matter in outdoor areas and become ineffective. Furthermore, spraying individuals with this chlorinating agent can cause eye and skin irritation [2]. In this context, the toxicity of NaOCl solutions varies with the concentration of NaOCl, in which a 0.1% NaOCl solution is the recommended concentration [38]. However, high concentrations of this disinfection agent can corrode mucous membranes (e.g., mouth, stomach, and respiratory tract), cause skin irritation, damage, and burn, gastrointestinal injury, and corneal epithelium burns [42]. Moreover, the dramatic increase in the use of disinfectants, including NaOCl and HOCl, due to the current pandemic, suggests an increase in resistance of bacteria present in the everyday human environment to these disinfectants. In addition to its use as disinfectant, HOCl can also be applied as a topical antiseptic agent for wound healing [43,44,45].

**Figure 1:**
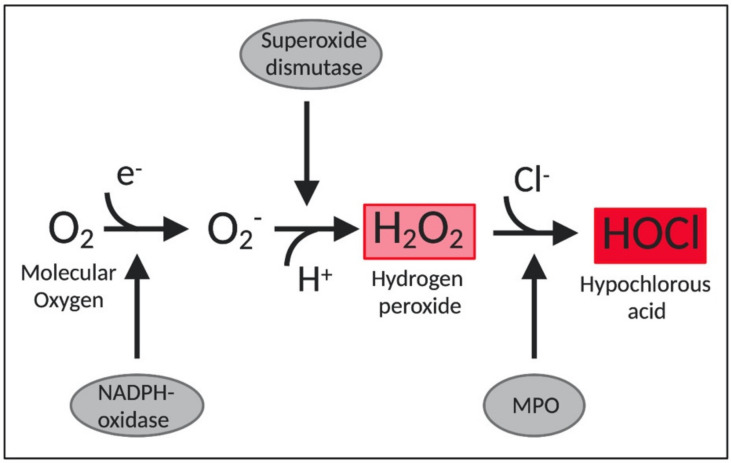
Schematic representation of HOCl production in neutrophils. MPO: myeloperoxidase. O_2_^−^: superoxide.

Importantly, HOCl is also one of the main oxidants produced by neutrophils and thereby indispensable for an effective innate immune response [46,47]. Neutrophils destroy invading pathogens by ingesting them through phagocytosis and by the release of antimicrobial proteins, oxidants, and digestive enzymes [46]. Furthermore, stimulated neutrophils also participate in a respiratory burst, which is accompanied by an increase in oxygen consumption and the consequent production of ROS by the enzyme NADPH-oxidase [43]. This enzyme complex assemblies in the plasma membrane and transfers electrons from NADPH to the molecular oxygen, forming superoxide (O_2_^•−^). Then, the enzyme superoxide dismutase converts O_2_^•−^ to hydrogen peroxide (H_2_O_2_), which serves as a precursor to several toxic species [46]. Of these, HOCl is produced from the reaction between H_2_O_2_ and chloride anion (Cl^−^), mediated by a heme enzyme called myeloperoxidase (MPO) [47] (Figure 1). Approximately 70% of H_2_O_2_ produced in neutrophils is converted into HOCl by myeloperoxidases [48]. HOCl is considered an extremely powerful oxidant. It is estimated that 10^6^ stimulated neutrophils are capable of producing approximately 0.2 μmol of HOCl during a 2-h incubation. In a matter of milliseconds, 0.2 μmol of HOCl is enough to destroy 150 million *E. coli* cells [49].

### 2.1. The Potent Antimicrobial Effects of HOCl

**Figure 2:**
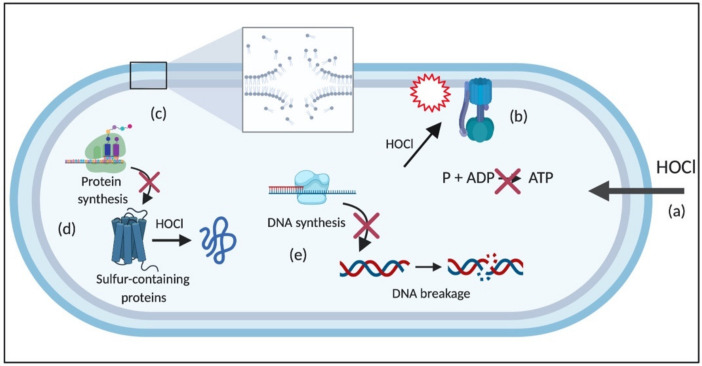
Schematic of HOCl targets in Gram-negative bacterial cells. (**a**) HOCl easily penetrates the bacterial cell due to its neutrality and attacks (**b**) several membrane components and processes, including transporters and proteins, such as ATPase, which disrupts ATP production (**c**) lipids, causing, for example, loss of membrane stability, (**d**) protein synthesis and proteins, especially the sulfur-containing ones, and (**e**) DNA, leading to DNA breakage and impairment of DNA synthesis. HOCl: hypochlorous acid; P: phosphate; ADP: adenosine diphosphate; ATP: adenosine triphosphate.

The potent antimicrobial activity of HOCl is due to its interaction with several cellular structures, as exemplified in Figure 2 for Gram-negative bacteria. Specifically, it rapidly undergoes reaction with nucleophilic structures, such as hemes and porphyrins, iron-sulfur proteins, purine and pyrimidine bases, sulfhydryl groups, amines, and amino acids [50].

Due to its electrical neutrality compared to ^−^OCl, HOCl can easily penetrate the cell wall and membrane of bacterial cells through passive diffusion, which results in multiple negative effects on cellular macromolecules and membrane-related processes [32]. Its reaction with membrane components causes damage to membrane proteins responsible for energy transduction and transport, leading to rapid ATP hydrolysis [51]; disrupts the cytoplasmic membrane-bound systems involved in fermentative and oxidative production of ATP, especially by oxidizing the F1 complex of the enzyme ATPase [52,53]; and inhibits metabolite and protein transport [54,55,56].

The reaction of HOCl with proteins and amino acids results in protein fragmentation due to the cleavage of peptide bonds, and protein unfolding, which leads to the irreversible aggregation of essential bacterial proteins, and consequent bacterial death [50]. The extent of these modifications depends on the concentration of HOCl and the structure of the protein [57]. For instance, sulfur-containing molecules, such as cysteine and methionine, are more susceptible to HOCl than other amino acids [58]. HOCl also suppresses bacterial growth by inhibiting protein and DNA synthesis due to its interaction with proteins involved in translation and transcription [55,59]. In addition, the reaction of HOCl with NH groups of nucleotides generates reactive chloramines, leading to the rupture of hydrogen bonds and the formation of nitrogen-centered radicals, which causes the dissociation of DNA double-strands [32,60]. Moreover, the reaction of HOCl with lipids generates chlorohydrin intermediates, which are more polar than regular lipids. The formation of these molecules can increase the permeability of the membrane and cause the loss of membrane function and structure [32] (Figure 2). The detailed reactions of HOCl with cellular molecules are described in the following sections.

Furthermore, the production of HOCl by neutrophils intensifies the damage caused by ROS to bacterial cells. It happens because HOCl inactivates or depletes bacterial oxidative defenses, such as antioxidants and oxidative enzymes. Then, the cells are not able to adequately respond to endogenously produced ROS [61]. As a result, ROS accumulates within the bacterial plasma membrane and can diffuse into the cytoplasm [32]. Also, the release of cellular iron, triggered by exposure to HOCl [62], can lead to a Fenton reaction between the released iron and H_2_O_2_, generating ·OH [32]. Besides that, ·OH is also produced via the reaction between HOCl and iron, which occurs three times faster than the Fenton reaction [63].

#### 2.1.1. Reaction of HOCl with Sulfur-Containing Amino Acids

**Figure 3:**
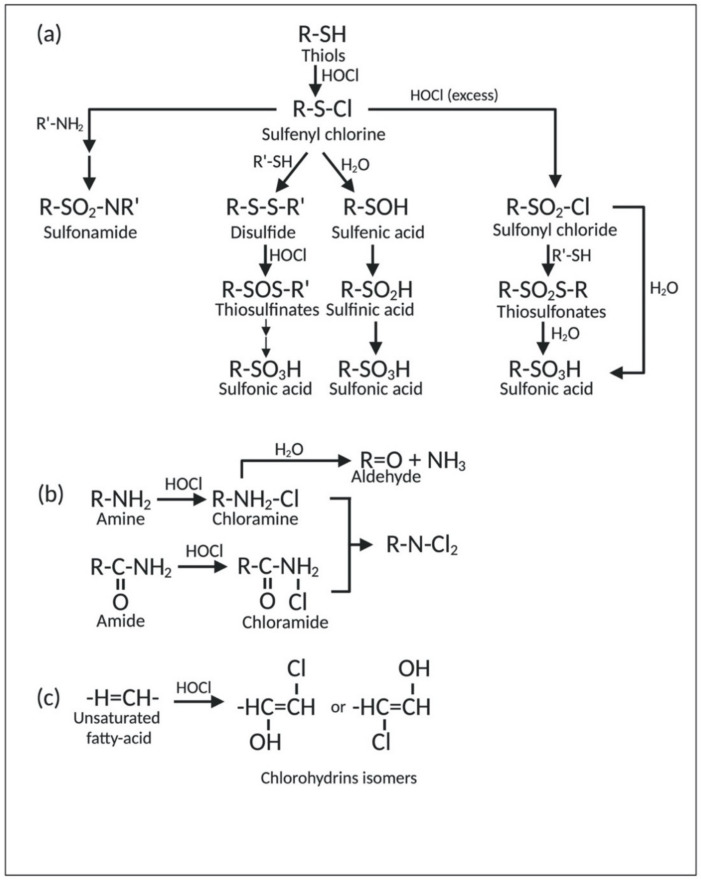
Reaction of HOCl with (**a**) thiol-containing compounds, (**b**) amine and amide, and (**c**) unsaturated fatty acids.

Proteins, the most abundant cellular molecules, are the main target of HOCl. In this context, sulfur-containing compounds, such as cysteine, methionine, and glutathione, have the highest degree of reactivity with HOCl compared to other biological molecules [64]. The reaction between thiol-containing compounds (R-SH), such as cysteine, and HOCl yields disulfides (R-S-S-R′) and sulfonic acids (R-SO_3_H). The initial step of this reaction is the chlorination of thiol-containing compounds (R-SH), which produces the sulfenyl chloride (R-S-Cl) intermediate. This intermediate can further undergo three different reaction pathways based on the chlorinating conditions. The first pathway yields sulfonic acid (R-SO_3_H) via sulfenic (R-SOH) and sulfinic (R-SO_2_H) acid intermediates. The second pathway first produces disulfide (R-S-S-R′) via the reaction with other thiol-containing compounds. Disulfides (R-S-S-R′) can then be converted into the oxygenated disulfide derivatives, namely thiosulfinates [R-S(O)-R′] or thiosulfonates [R-S(O_2_)-S-R′]. Eventually, these oxygenated disulfide derivatives are hydrolyzed to form sulfonic acid (R-SO_3_H). In the last pathway, sulfenyl chloride (R-S-Cl) is converted into sulfonyl chloride (R-SO_2_-Cl) in excess of HOCl. Sulfonyl chloride (R-SO_2_Cl) can be further converted into thiosulfonates [R-S(O_2_)-S-R′] upon reaction with thiols (R-SH). Both sulfonyl chloride (R-SO_2_Cl) and thiosulfonates [R-S(O_2_)-S-R′] can be hydrolyzed to form sulfonic acid (R-SO_3_H) [35]. Generally, the irreversible formation of sulfonic acid (R-SO_3_H) either inhibits protein formation or targets them for degradation [65]. In addition to the reactions mentioned above, sulfonyl (R-SO_2_Cl) and sulfenyl (R-S-Cl) chlorides can also react with amino compounds (R′-NH_2_) to form sulfenamide (R-S-N-R′), sulfinamide (R-SO-N-R′), as well as irreversible sulfonamide linkages (R-SO_2_-N-R′) [35,66]. Furthermore, sulfenyl chlorides (R-S-Cl) can decompose into thiyl radicals (R-S) under high temperature, presence of metal ions, or UV light. Lastly, sulfides (R-S-R′) can also be attacked by chlorine, which subsequently leads to the formation of sulfuryl-containing (R-SO_2_-R′) compounds upon hydrolysis [35]. A schematic representation of the reaction of HOCl with thiol-containing compounds is illustrated in Figure 3a.

Methionine is also a target of HOCl. The oxidation of the thioether group on the side chain of methionine produces methionine sulfoxide (Met-SO) and methionine sulfone (Met-SO_2_). Although the formation of methionine sulfoxide (Met-SO) can be reversed by the methionine sulfoxide reductase family of enzymes, oxidation of methionine-to-methionine sulfone (Met-SO_2_) is considered irreversible. Moreover, HOCl can also convert free methionine and methionine residues at the N-terminus of the proteins into dehydromethionine [67]. The formation of this cyclized product is considered irreversible [68].

#### 2.1.2. Reaction of HOCl with Aromatic Amino Acids

The reaction of HOCl with aromatic amino acids is another important effect of HOCl stress. For instance, HOCl undergoes reaction with tryptophan on proteins, which might lead to irreversible protein unfolding and enzyme inactivation [67]. Furthermore, HOCl can also chlorinate the side chain of tyrosine. Chlorinated products of this reaction (3-chlorotyrosine and 3,5-dichlorotyrosine) have been suggested as biomarkers of protein damage resulting from HOCl and chloramine derivatives [67,69]. Chloramines are disinfectants that are commonly used to treat drinking water.

#### 2.1.3. Reaction of HOCl with Nitrogen-Containing Compounds

HOCl rapidly reacts with nitrogen-containing compounds (particularly amines and, to a lesser extent, amides), yielding chlorinated derivatives, including chloramines (R-N(R′)-Cl) and chloramides (R-C(O)-N(R′)-Cl) (Figure 3b). These compounds can be further oxidized to form dichlorinated species (R-N-Cl_2_). Although capable of oxidizing other molecules, all of the compounds mentioned above are milder than HOCl and react more slowly [67]. Moreover, chloramines can decompose into corresponding aldehydes or form nitrogen radicals in a metal-catalyzed reaction [70]. In addition, the chlorination of nitrogen-containing moieties can occur at the α-amino group of amino acids, on the N-terminus of peptides and proteins, and at the nucleophilic centers of protein side chains (e.g., lysine, arginine). On the other hand, chloramides can be generated at the peptide bonds of proteins and, in excess of HOCl, on the side chains of asparagine and glutamine, which contain amide groups [67]. The formation of chloramines and protein carbonylation (aldehyde formation) interferes with protein folding and leads to protein aggregation [71].

In nucleotides, the main targets of HOCl are primary and secondary amino groups of guanosine, cytosine, adenosine, uridine, and thymidine of DNA and RNA molecules. This reaction also results in the formation of chloramines, which destabilize the DNA strand and causes their breakage [60].

#### 2.1.4. Reaction of HOCl with Lipids

The reaction between HOCl and acyl chains of unsaturated fatty acids primarily results in the production of chlorohydrins [72] (Figure 3c). The formation of these lipid chlorohydrins is involved in cell lysis, pro-inflammatory effects, and toxicity [73]. However, in phospholipids containing a primary amino group, HOCl preferably reacts with the nitrogen-bearing group to produce chloramines [72]. In addition, HOCl can also induce lipid peroxidation by radicals derived from reactions of this disinfectant with amines and organic hydroperoxides [72,74].

## 3. Adaptive Response of Gram-Negative Cells to HOCl

The damage caused by oxidative stress to bacterial cells is often irreversible. Therefore, microorganisms must sense and adapt to environmental conditions to maintain their cellular homeostasis. While the adaptive response to ROS has been extensively studied, the knowledge about how bacteria sense and respond to the damage caused by HOCl is still limited, and many questions regarding the adaptive regulatory pathways adopted by them remain unsolved. Moreover, several defense mechanisms involved in the adaptive response to ROS have also been described to be involved in HOCl resistance.

**Figure 4:**
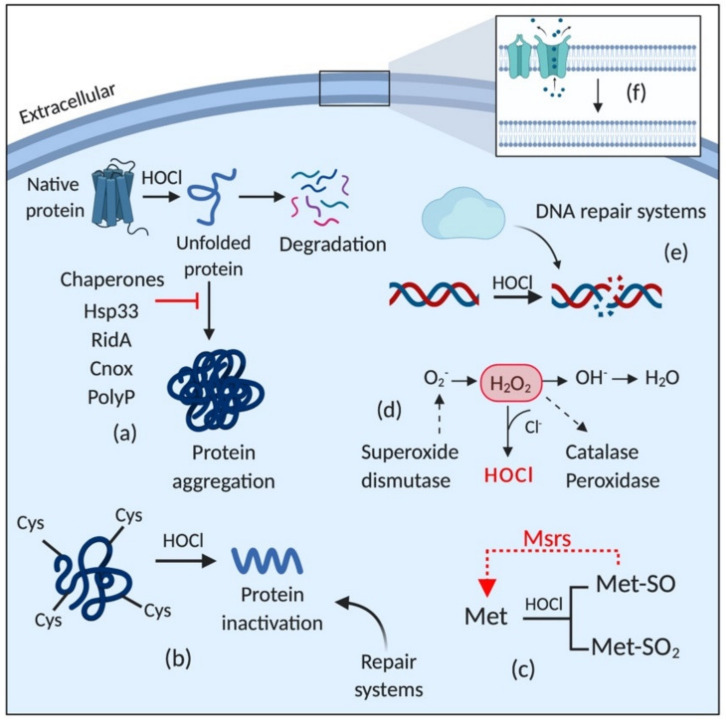
Schematic representation of the stress response triggered by HOCl exposure. HOCl activates (**a**) chaperones; induces the expression of (**b**) protein repair systems, especially for those in which cysteine and (**c**) methionine are the main target amino acids; (**d**) detoxifying enzymes; (**e**) DNA repair systems; and (**f**) induces changes in membrane properties, such as downregulating porins, reducing permeability and increasing hydrophobicity. HOCl: hypochlorous acid; H_2_O_2_: hydrogen peroxide; Cys: cysteine; Met: methionine; Met-SO: methionine sulfoxide; Met-SO2: methionine sulfone; Msrs: methionine sulfoxide reductases.

Figure 4 depicts a general overview of the adaptive responses of Gram-negative bacteria to HOCl (for more details, see [70]). HOCl induces the expression of several detoxifying enzymes, including catalases [75], peroxidases [76], and superoxide dismutase [77]; activates chaperones [78,79]; DNA [80] and protein [75,77] repair systems, including Fe-S clusters repair; upregulates the expression of methionine sulfoxide reductases (Msrs) [81]; and induces changes in the membrane, such as increasing hydrophobicity, reducing permeability, and decreasing the amount of porins [82,83].

### 3.1. Chaperones: The Immediate Response against HOCl-Induced Stress

In order to adapt to the damage caused by oxidative stress, microorganisms must alter their metabolism and induce several response mechanisms controlled by transcriptional regulators [84]. However, the complex regulation of transcriptional sensors is a slow process compared to the high reactivity of HOCl with sidechains of amino acids [85]. For instance, while the reaction rate of HOCl with methionine and cysteine is approximately 3 × 10^7^ M^−1^s^−1^, the expression of a stress response protein can take up to one hour [85,86,87]. Therefore, bacterial cells under HOCl stress immediately activate chaperones to cope with the proteome damage caused by this disinfectant [78]. Chaperones are heat-shock proteins that bind to other proteins and prevent their misfolding and the formation of lethal protein aggregates [88,89]. They are activated in response to the accumulation of protein unfolding intermediates [86].

HOCl substantially decreases the number of cellular proteins due to its high reactivity with proteins involved in transcription and translation and disrupts ATP synthesis by inhibiting the F1 complex of the enzyme ATPase [52]. It makes the action of ATP-independent chaperones, also called holdases, crucial for the efficient response against HOCl oxidative stress. In this context, three ATP-independent HOCl-sensing chaperones have been identified: Hsp33 (heat shock protein), RidA, and CnoX [78,86]. Hsp33 was identified two decades ago as the first chaperone involved in the HOCl-stress response and is the most frequently studied [78,85]. Hsp33, first identified as HslO, is specifically activated in response to the generation of unfolding proteins by HOCl. However, it can also be activated by other oxidizing agents to a lesser extent, such as H_2_O_2_ and nitric oxide, and protein unfolding conditions, such as oxidative heat stress [90].

Hsp33 is a heat shock protein that has six cysteine residues, four of which coordinate a zinc center [91]. Under nonstress conditions, the cysteine residues are in a reduced state, and this chaperone is inactive. However, exposure to oxidizing agents induces the release of the zinc ion and the formation of two intramolecular disulfide bonds in the Hsp33 molecule, inducing its partial unfolding. These modifications lead to the formation of the dimeric activated Hsp33. The unfolded region binds to unfolded proteins, avoiding their non-specific aggregation [43,92]. When oxidative stress is under control, Hsp33 releases the unfolded protein and returns to the reduced state. Then, ATP-dependent foldase systems, such as DnaK/J/GrpE, refold the released proteins [93]. Several studies have demonstrated the role of Hsp33 in bacterial protection against HOCl oxidative stress in *E. coli* [50,94,95,96], and in *Vibrio cholerae* [90]. Hsp33 is activated in response to the increase in protein unfolding caused, for example, by oxidative stress and heat [97]. Furthermore, under oxidative stress conditions, Hsp33 binds to EF-Tu, a protein elongation factor substantially sensitive to HOCl, preventing its unfolding and unspecific degradation in *V. cholerae* [90] and *E. coli* [94]. It suggests that the stabilization of EF-Tu in *V. cholerae* is essential for the stress resistance of this bacteria.

Another class of proteins involved in the HOCl-stress response is the YjgF/YER057c/UK114 family, composed of eight subfamilies: Rid1-Rid7 and RidA. RidA is a enamine/imine deaminase that naturally synthesizes branched-chain amino acids [78]. Studies have shown that when exposed to HOCl stress, RidA is chlorinated and loses its deaminase activity and plays an important role as a chaperone [98]. Müller et al. (2014) showed that RidA prevented protein aggregation and that RidA mutants are more sensitive to the oxidative effect of HOCl than the wild type. Furthermore, they showed that the incubation of RidA with H_2_O_2_ and diamine did not reduce its holdase activity, indicating that RidA acts as a HOCl-specific chaperone under oxidative stress. The activation of RidA is similar to the process that occurs in Hsp33; however, it does not involve cysteine activation. RidA is activated by chlorination of positively charged amino acids, such as lysine and arginine, which increases its hydrophobicity, and induces the binding to unfolded proteins [99].

CnoX, initially called YbbN, is a dual function chaperodoxin widely conserved in Gram-negative bacteria that prevents protein aggregation and protein oxidation [100]. The role of CnoX as a chaperone was first described in 2018 by Goemans and collaborators [101]. CnoX is composed of two complementary domains: an N-terminal thioredoxin (Trx) and a C-terminal tetratricopeptide repeat (TPR) domain [102]. The process of activation of the holdase activity of CnoX is similar to the process that occurs in RidA. HOCl chlorinates several amino acid residues in the TPR domain, increasing the hydrophobicity of the protein and its affinity to unfolded proteins. On the other hand, the chlorination of Cys63 of the Trx domain induces the formation of disulfide bonds with other proteins, protecting them against HOCl oxidation [100,102]. Like Hsp33, CnoX cooperates with DnaK/J/GrpE refolding systems, but it is the only identified chaperone that also transfers its substrates to the foldase system GroEL/ES [101]. Goemans and collaborators [101] proved that *E. coli* CnoX has potent chaperone activity under HOCl stress. They showed that CnoX can interact with several substrates and is readily activated by exposure to HOCl, but its holdase activity is not induced by H_2_O_2_ and diamide. In addition to its chaperone activity, CnoX also protects cysteine residues of HOCl substrates by forming disulfide bonds with them [101].

In addition to the chaperone holdases mentioned, Groitl and collaborators [79] showed that inorganic polyphosphate (polyP) is overproduced in *P. aeruginosa* under HOCl stress and that the absence of this molecule causes massive protein aggregation. It indicates that under stress conditions, polyP acts as a chaperone [79]. PolyP is a inorganic molecule involved in several cellular processes, including phosphate and energy storage, metal chelation, and pH buffering [103]. The role of polyP as a chaperone holdase was first described in 2014 by Gray and collaborators [103]. PolyP is linked to the resistance of *E. coli* to HOCl because it stabilizes unfolded proteins and prevents their aggregation [103]. Like Hsp33, the accumulation of polyP is directly regulated by HOCl and does not require transcription and ATP, which represents a quick response against HOCl stress. When HOCl reacts with proteins and forms protein aggregates, the conversion of ATP to polyP is induced through the inactivation of PPX (polyP-degrading exopolyphosphatase). Then, polyP complexes with unfolded proteins. Once the oxidative stress has been controlled, PPK (ATP polyphosphate kinase) degrades polyP and release ATP [104,105]. Although phosphate does not react with HOCl, the conversion of ATP into polyP is considered the primary cause of phosphate starvation [103], which is linked to the increased production of methylglyoxal. This toxic electrophile can react with DNA, RNA, and proteins and cause cell damage [106].

### 3.2. Transcriptional Regulators

**Figure 5:**
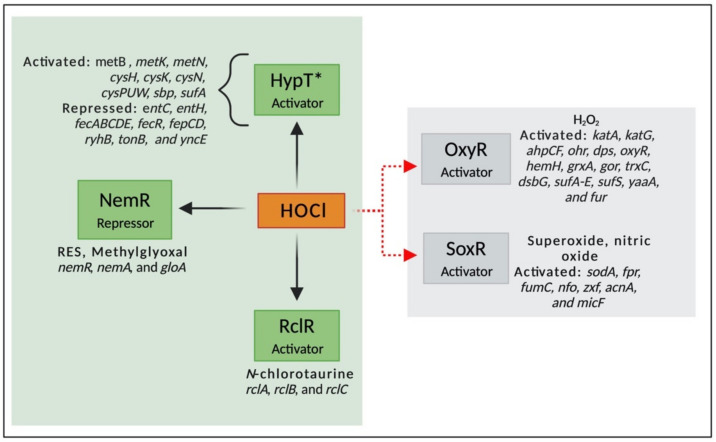
Activation of transcriptional factors by HOCl stress (green area). *HypT is the only regulator known to be specifically activated by HOCl. Its activation induces the expression of genes involved in cysteine, methionine, and sulfur biosynthesis, and downregulates genes involved in iron acquisition and homeostasis. NemR, a regulator responsive to HOCl, RES, and methylglyoxal, upregulates genes involved in glyoxylase and *N*-ethylmaleimide synthesis. RclR is activated by HOCl and *N*-chlorotaurine and expresses a flavoprotein disulfide reductase, an uncharacterized periplasmic protein, and transmembrane quinone-binding protein. The gray area shows the genes regulated by OxyR and SoxR, the most studied ROS-sensors. HOCl: hypochlorous acid; H_2_O_2_: hydrogen peroxide.

Transcriptional factors are proteins that bind to DNA promoter sequences and regulates the transcription of several genes, stimulating or repressing gene expression. They are activated by different molecules and pathways; for example, some transcriptional factors are activated by the presence of oxidizing agents and are essential for bacterial survival under oxidative conditions [107,108]. While the mechanisms involved in response to ROS are well characterized, the knowledge about regulatory networks involved in RCS is still limited. Moreover, sensor proteins that respond to HOCl are often also responsive to other stressors, especially ROS. In this context, very few transcriptional regulators that specifically respond to HOCl have been identified. These include three HOCl-sensing transcriptional regulators described in *E. coli*, HypT, RclR, and NemR [109]. In addition, the knowledge about how the genes controlled by these transcriptional factors contribute to bacterial survival under HOCl is still minimal [110]. In this review, we summarize the HOCl-specific transcriptional regulators and two widely studied regulators (OxyR and SoxR) that are involved in ROS response but have also been described to be involved in HOCl resistance in Gram-negative bacteria (Figure 5).

The first transcriptional factor described that specifically responds to HOCl, YjiE, belongs to the family LysR-type transcriptional regulator (LTTR) [111]. LTTR, the largest family of transcriptional regulators described in prokaryotes, is composed of a helix-turn-helix (HTH) on its DNA binding domain (DBD) and a regulatory domain (RD) that can activate or repress gene expression [112]. It regulates the transcription of several genes, including those involved in adherence [113], efflux pump [114], antibiotic resistance [115], biofilm formation [116], and oxidative stress response [117].

YjiE was renamed HypT (hypochlorite-responsive transcription factor) due to its specificity to HOCl. Gebendorfer and collaborators (2012) found that HypT is a conserved transcription factor that confers resistance to HOCl but not to H_2_O_2_ and diamide [111]. The high reactivity of HOCl with methionine residues in comparison to H_2_O_2_ explains the specificity of this regulator to HOCl [118]. HypT upregulates genes involved in sulfur, methionine, and cysteine biosynthesis and down-regulates genes associated with iron acquisition and homeostasis [111]. Iron is essential in many metabolic pathways (e.g., respiration and enzyme activity). However, it can be toxic to cells by inducing the production of ROS through the Fenton reaction [119]. Thus, the lower acquisition of iron could be essential to keep iron homeostasis under oxidative stress response. Furthermore, because the main targets of HOCl are sulfur-containing compounds, mainly cysteine and methionine, it is not surprising that HOCl decreases the intracellular levels of these molecules, leading to the up-regulation of genes involved in their biosynthesis.

HypT is a transcriptional regulator as sensitive to HOCl as the chaperone Hsp33 but it is activated by the oxidation of three Met residues (Met123, Met206, and Met230) to methionine sulfoxide rather than by the oxidation of cysteine, as observed in Hsp33 [120]. However, the cysteine residues in HypT are essential for the stability of the molecule. The substitution of Cys150 of the HypT structure by serine altered inter- and intramolecular contacts in the HypT molecule, which destabilized its structure. Furthermore, under oxidative stress that is not sufficient to activate HypT, Cys4 is oxidized and prevents the interaction of HypT with DNA, which avoids the unnecessary expression of HypT. Therefore, Cys4 is considered a check point for the activation of this transcriptional regulator [121]. It indicates that though they are not involved in the activation of HypT, they play an important role in stability [121]. HOCl causes changes in the RD of the oxidized HypT, inducing a counter-rotation of DBD. Then, HypT binds to two separated DNA motifs in the promoter *fhu*A, forming a DNA loop and repressing gene expression [118]. HypT can form dimers, tetramers, and dodecamers, which can dissociate under stress conditions. The dodecamer structure is inactive, and when HOCl activates HypT, its dodecamers reversibly dissociate into tetramers and become quickly available for methionine oxidation [111,122]. Furthermore, Drazic and collaborators (2014) showed that the presence of NaCl and arginine in the medium increased the thermal stability of HypT, suggesting that these compounds would also stabilize the protein against the effect caused by HOCl [122]. Indeed, they found that NaCl and arginine compromised the quaternary structure of HypT and induced the dissociation of the dodecamer to further stabilize its dissociated oligomers [122]. HypT can be inactivated by MsrA and MsrB proteins [120] that belong to the methionine sulfoxide reductases (Msrs) and reduce methionine sulfoxide back to methionine [123].

The transcriptional regulator RclR is a member of the AraC family and specifically responds and promotes bacterial survival under stress caused by HOCl and *N*-chlorotaurine [108,124]. *N*-chlorotaurine is formed by the reaction of HOCl with taurine, a semi-essential amino acid widely found in the cytoplasm of neutrophils [125]. Parker et al. (2013) found that HOCl and *N*-chlorotaurine significantly induced the transcription of *rclB* (100–500-fold compared to wild type) in *E. coli*. However, the addition of ROS (H_2_O_2_, *tert*-butyl hydroperoxide, and methyl viologen), reactive electrophile species (RES) (diamide, methylglyoxal, and *N*-ethylmaleimide), reactive nitrogen species (RNS) (nitric oxide and peroxynitrite), as well as other RCS (chloroform and trichloroacetic acid) did not induce *rclB*. These findings suggest that RclR is an HOCl-sensitive transcriptional regulator [108].

RclR, formally known as YkgD, is conserved in Gram-negative bacteria and activated by the oxidation of two cysteine residues (Cys-21 and Cys-89). These oxidized cysteines form a disulfide bond and stabilize the RclR oxidized molecule. The activation of RclR induces the activation of three genes involved in HOCl adaptive response: *rclA* (flavoprotein disulfide reductase), *rclB* (uncharacterized periplasmic protein), and *rclC* (transmembrane quinone-binding protein) [109]. The deletion of the genes *rclA* or *rclR* decreased by 30% the survival rate of *Salmonella* in macrophages containing sub-lethal concentrations of HOCl, suggesting the role of RclA in the HOCl resistance [109]. In *E. coli*, mutants lacking any of the four genes were more sensitive to HOCl than the wild-type, indicating that *rclA*, *rclB*, *rclC*, and *rclR* are closely involved in the adaptive response to HOCl stress [108]. RclA is a HOCl-resistant copper (II) reductase that reduces Cu (II) to Cu (I). The reduction of Cu (II) to Cu (I) restricts the production of the highly reactive Cu (III), which is produced by the reaction of HOCl with Cu (II) and can cause severe damage to cells [126]. The molecular modifications responsible for the upregulation of *rcl* genes, as well as the role of RclB and RclC in HOCl resistance, are still unclear [124,126].

The third transcriptional regulator described to be involved in the HOCl stress response is the NemR (*N*-ethylmaleimide (NEM)-specific transcriptional regulator) repressor [109]. NemR is a member of the TetR family and is responsible for the expression of a glycoxylase (GloA) and *N*-ethylmaleimide reductase (NemA) [124]. It is activated in response to RES (e.g., quinones, glycoxals, and *N*-ethylmaleimide (NEM)) [127], methylglyoxal (MGO), and RCS [109]. One of the effects of the exposure to HOCl is the accumulation of dihydroxyacetone phosphate (DHAP) and the depletion of phosphate pools, which is associated with the formation of MGO [103]. MGO, a toxic electrophile produced under unbalanced metabolism of sugar, is converted into D-lactate by glycoxylases [128], suggesting that NemR is essential for bacterial survival under oxidative stress. NemA is associated with detoxification of quinones, ketones, and aldehydes, including MGO [129].

NemR has six cysteine residues (Cys21, Cys98, Cys106, Cys116, Cys149, and Cys153) responsible for the adaptive response to electrophiles and RCS. While Cys106 is involved in the adaptive response to RCS, Cys21, and Cys116 are involved in the response to electrophiles [130]. The activation of NemR by electrophiles is mediated by the formation of disulfide bonds at Cys21 and Cys116 residues in vivo and in vitro [127]. On the other hand, the HOCl response is mediated by the formation of a sulfonamide bond between Cys106 and Lys175, leading to a sulfonamide thiol switch. The oxidation of cysteine residues by HOCl produces sulfenyl chlorides, which are highly reactive with lysine, explaining the specificity of NemR to HOCl [104,131]. In the absence of stress conditions, NemR is bound to the DNA and represses transcription. Conversely, under stress conditions, the oxidation of Cys116 causes the dissociation of NemR from the DNA and induces the expression of *gloA* and *nemA* [124].

Besides the HOCl-sensing transcriptional regulators mentioned above, several redox sensors that are involved in ROS stress response have also been described to be involved in HOCl resistance in Gram-negative bacteria. These shared defense mechanisms have been previously summarized in excellent reviews [70,132,133]. Here, we will provide just a brief overview of the most common and widely studied regulators involved in H_2_O_2_ and superoxide response (OxyR and SoxR) [70,108].

OxyR is a well-described H_2_O_2_-sensing transcriptional regulator that belongs to the LTTR family. It is involved in the expression of catalases, hydroperoxide reductase, DNA-binding protein (Dps), heme biosynthesis, repression of iron and manganese transport, and Fe-S repair [133,134]. Despite its role in H_2_O_2_ resistance, OxyR has also been associated with HOCl stress response in *Xanthomonas campestris* [134], *E. coli* [77], and *Salmonella* [135]. The mechanisms of activation of OxyR by HOCl is not well understood; however, this transcription factor is activated by H_2_O_2_ through the oxidation of the Cys199, which forms a disulfide bond with Cys208 and causes conformational changes in the OxyR structure. In the absence of oxidizing agents, OxyR has a dimeric form, which binds to two DNA-binding regions. On the other hand, in an oxidized state, OxyR has a tetrameric conformation and binds to four DNA regions. These conformational changes alter the specificity of OxyR to target promoters, altering its interaction with RNA polymerase, thereby inhibiting transcription [133,136].

While OxyR is an H_2_O_2_ sensor, SoxR belongs to the MerR family and is activated by superoxide compounds, such as paraquat, and nitric oxide [137]. It was believed that SoxR was insensitive to H_2_O_2_; however, now it is known that this redox sensor is also activated by this ROS. For instance, Semchyshyn et al. (2005) showed that H_2_O_2_ induced the expression of superoxide dismutase and glucose-6-phosphate dehydrogenase in *E. coli*, which are proteins regulated by this transcription factor [138]. The oxidation of SoxR induces the activation of a second redox sensor, SoxS, inducing the transcription of several genes, including manganese superoxide dismutase, ferredoxin, flavodoxin-NADP^+^ reductase, fumarase C, endonuclease IV, glucose-6-phosphate dehydrogenase, aconitase, and a regulatory RNA (*micF*) [139,140]. SoxR is a dimeric protein that contains 2Fe-2S centers that are oxidized and activated by redox compounds. Additionally, studies have proved that this transcription factor is also involved in HOCl resistance [61,141]. Mutations on the *soxS* gene of *Salmonella typhimurium* strains produced mutants more sensitive to the effect of NaOCl [142].

Other redox sensors primarily known to be involved in ROS resistance have also been described in HOCl response in Gram-negative bacteria. These include the two-component system aerobic respiration control (ArcAB) [82,143,144], SylA [145] and OhrR [134], which are members of the MarR family, PerR, which belongs to the Fur family [70], and ComR, controlled by the TetR family [70].

### 3.3. Formation of Biofilms as an Adaptive Response to HOCl

The formation of biofilms is a survival strategy adopted by bacterial cells in response to the damage caused by environmental stressors, such as sub-lethal concentrations of antimicrobial and oxidizing agents [146,147,148]. The molecular mechanisms involved in biofilm stimulation by these stressors have not been elucidated. However, it is suggested that sub-inhibitory concentrations of antimicrobial agents can induce the formation of biofilms by provoking the release of intracellular material, especially extracellular DNA (eDNA), which is an essential component of the EPS matrix, and inducing transcriptional and cell morphology changes, which can, for example, enhance cellular adhesion [148].

Several studies have shown the antibiofilm effect of HOCl on Gram-negative bacteria [149,150,151]. However, bacteria exposed to sub-lethal concentrations of this disinfectant have recovered from the damage caused by HOCl, being able to form robust biofilms [147,152,153]. Lin et al., (2017) treated isolates of *Klebsiella*, *Pseudomonas*, *Flavobacterium*, and *Sphingomonas* biofilms with sub-lethal and lethal concentrations (0, 0.125, 0.25, 0.5, 1, 2, 5, and 10 mg/mL) of NaClO, and showed that, initially, increasing concentrations of this disinfectant decreased the bacterial burden. However, 30 min after the treatment, all four biofilms persisted, suggesting a bacterial adaptation to NaClO. Furthermore, an increased number of cell clusters was also detected after the treatment with the highest concentration (10 mg/mL) of NaClO, suggesting that chlorine depleted the EPS matrix, causing splitting of the biofilm [154]. NaOCl disrupts the extracellular polymeric matrix of biofilms, causing a significant reduction of this structure in comparison to H_2_O_2_ [155]. In this context, the dispersal of single cells (or clusters of cells) is an essential step in biofilm formation since it contributes to their spread.

The mechanisms involved in biofilm stimulation, as well as the adaptative responses to HOCl, remain to be characterized and may be less predictable in biofilms than in planktonic cells due to the complexity and the metabolic diversity of these structures [84]. For instance, previous exposure to sub-inhibitory concentrations of NaClO induced the formation of robust biofilms in *E. coli* apparently by increasing the cell hydrophobicity and changing the outer membrane properties [152]. Cell hydrophobicity is crucial for bacterial adhesion and host invasion. These changes in membrane properties are due to the interaction of HOCl with membrane proteins and lipids [156,157]. Additionally, exposure to sub-lethal concentrations of HOCl induced a morphological change in *Salmonella* Heidelberg from a smooth to rugose morphology. In this context, rugose cells formed more robust biofilms in plastic and stainless-steel surfaces in comparison to non-adapted cells [158]. The change in colony morphology is known to be an adaptative response to oxidative stress, in which rugose cells are more resistant to osmotic, temperature, and oxidative stress, as well as present higher ability to form biofilms [159]. Also, rugose variants express high levels of RbmA, which is essential for the maintenance of the rugose morphology, and the development of biofilm architecture [159].

Moreover, a higher amount of EPS and an increased expression of the genes involved in environmental stress response (*envZ* and *csrA*), curli synthesis (*csgA*, *csgB*, and *csgC*), cellulose formation (*bscE*), biofilm regulators (*csgD* and *hns*), and stress resistance and biofilm formation (*rpos*), were detected in rugose *S.* Heidelberg biofilms in comparison to smooth cells. In this case, the adaptative response of *S.* Heidelberg biofilms to NaClO could be due to the increased production of EPS, which protects the cells against the disinfectant, and the expression of cellulose and curli, which is important for biofilm attachment [160]. The EPS matrix provides not only a physical but also chemical protection against environmental stressors, which is crucial for adaptation in hostile conditions. For instance, it protects the cells by providing enough time for them to change their metabolism and limit the intracellular production of ROS [84].

Strempel et al. (2017) found that the early steps of *P. aeruginosa* cells attachment and, the consequent formation of biofilm upon exposure to NaClO is enhanced by the overexpression of a diguanylate cyclase (DCG) (PA3177) and increased intracellular levels of cyclic-di-GMP (c-di-GMP), suggesting the role of this second messenger in NaClO stress response [161]. C-di-GMP, a second messenger ubiquitous in bacteria, is involved in the transition from the motile lifestyle to a sessile phenotype by controlling processes involved in the production of adhesins, pili, fimbriae, EPS, biofilm formation, cell surface changes, and oxidative stress resistance [162,163]. It is produced by DCGs and hydrolyzed by phosphodiesterases (PDEs). The synthesis of c-di-GMP is mediated by the two GDP domains (GGDEF) bound to a GTP molecule in each promoter in an antiparallel manner, which induces the formation of two intramolecular phosphodiester bonds [164]. On the other hand, PDEs have EAL or HD-GYP domains. The EAL-type PDE breaks down c-di-GMP in the presence of Mg^2+^ or Mn^2+^ and forms a linear 5′-phosphoguanylyl-(3′-5′)-guanosine (pGpG). The HD-GYP-type PDE hydrolyzes c-di-GMP in a two steps reaction, producing two GMP molecules. *P. aeruginosa* has five well-known DGCs involved in biofilm formation (WspR, SadC, RoeA, SiaD, and YfiN/TpbB) [165]. Although the role of c-di-GMP in oxidative stress has been described, the molecular mechanisms by which HOCl and other oxidizing agents activate DCG and induce the synthesis of c-di-GMP is not entirely elucidated [161,163,166].

Several cellular pathways are induced or repressed in response to exposure to oxidizing agents. *P. aeruginosa* wild type and the mutants Δ*rclX*, and Δ*rclR* were grown in the presence and absence of HOCl and HOSCN. All three strains were able to form biofilms in the absence of the oxidizing agents. However, the addition of 3.5 mM and 0.53 mM of HOCl and HOSCN, respectively, inhibited the formation of biofilms, suggesting thereby, that these genes are involved in the adaptation of *P. aeruginosa* to oxidative stress (see Section 3.2). RclR protects *P. aeruginosa* against oxidative stress by upregulating *rclX*, a putative peroxiredoxin [167]. RclR belongs to the AraC family and, along with HypT, is the first HOCl-sensing transcriptional regulator that responds specifically to HOCl, but do not respond to ROS and thiol-reactive compounds [132]. RclR is not only involved in HOCl-stress response in biofilms, but this transcriptional regulator is also crucial for the protection of motile bacteria against exogenous HOCl [108,167].

Transcriptomic analysis has been extensively conducted to evaluate the gene expression profile of bacteria under stress conditions. For instance, Peeters et al. (2010) conducted a transcriptomic analysis to evaluate the gene expression of *Burkholderia cenocepacia* biofilms in the presence of HOCl and H_2_O_2_. Upon exposure to 0.02% of HOCl for 5 min, 386 genes were upregulated, and 331 genes were downregulated. Among the genes involved in HOCl response, those involved in the regulation of scavenging enzymes and oxidative stress response (e.g., hydroperoxide resistance protein, alkyl hydroperoxide reductase, carboxymuconolactone decarboxylase, OsmC-like protein) were marked upregulated (37.2- to 75.2-fold increase in comparison to untreated biofilms). Also, genes encoding enzymes involved in DNA repair (recombinase A and Dps), repair of sulfur-cluster and iron-sulfur cluster containing proteins (e.g., aconitase B (*acnB*), cysteine desulfurase (*iscS*), ferredoxin (*fdx*), chaperones (*hscA* and *hscB*), and iron-sulfur cluster scaffold protein (*iscA*)), and motility (*flhB*, *fliF*, *flgF*, and *flgL*) were induced [168]. Some of these genes are directly involved in biofilm formation. For instance, flagella are known to be essential for cell adhesion in the early stages of biofilm formation [169]. Then, the upregulation of flagellar structures could induce the formation of robust biofilms, representing a protective strategy against HOCl stress.

**Figure 6:**
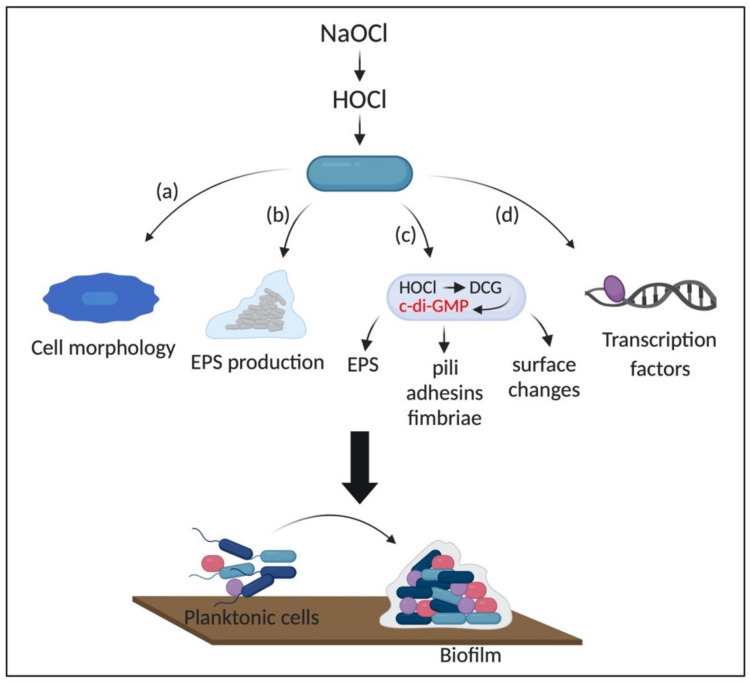
Possible mechanisms of HOCl biofilm stimulation in Gram-negative bacteria. HOCl induces (**a**) changes in cell morphology, which enhance the ability to adhere to surfaces; (**b**) increases the production of extracellular polymeric substance (EPS) matrix, increasing cell tolerance; (**c**) upregulates the production of c-di-GMP by DCGs; (**d**) regulates transcriptional regulators involved in HOCl-induced stress response. NaOCl: sodium hypochlorite; HOCl: hypochlorous acid; EPS: extracellular polymeric substance; DCG: diguanylate cyclase; c-di-GMP: cyclic-di-GMP.

Additionally, Lipus and collaborators (2019) evaluated the gene expression of *Pseudomonas fluorescens* biofilms after exposure to 0.6 mg/L of NaClO through RNA-seq analysis. They found that genes related to oxidative stress (e.g., *ohr*, *ahpC*, *ahpF*, *trxB*, *yedY*, and *katA*), multidrug efflux pumps and membrane stability (e.g., *mexE*, *terC*, *ssuF*, *copZ*, *potAB*, *fdpA*, and *araJ*), and transcription regulators (e.g., *araC*, *tetR*, *lysR*, *arsR*, and *iscR*) were upregulated. Conversely, genes involved in amino acid metabolism (*msdh*), energy production (e.g., *ydfG*), and membrane transport (e.g., *pbuE*, *kgtP*, and *ptsA*) were downregulated upon exposure to HOCl. The gene *ohr* encoding a hydroperoxide resistance protein was the most upregulated (30-fold increase in expression) [170]. The organic hydroperoxide resistance protein Ohr and alkyl hydroperoxide reductase are enzymes produced in response to organic peroxides, H_2_O_2_, and peroxynitrite by the organic peroxide-sensing repressor OhrR and OxyR regulator, respectively [171,172]. The role of OhrR and OxyR in the survival of Gram-negative bacteria under oxidative stress conditions have been extensively reported for planktonic cells [70,133,134]. In addition, catalases are enzymes that decompose H_2_O_2_, thereby representing an essential survival strategy to oxidative stress [173]. Furthermore, because HOCl promptly reacts with cysteine residues of amino acids, it is expected that repair systems would be activated, as observed in the upregulation of membrane repair systems (YedYZ and TrxB) [170]. We hypothesize that genes involved in amino acid metabolism could be downregulated to maintain a low intracellular concentration of these biomolecules. Moreover, since the primary targets of HOCl are side chains of amino acids, the low production of these molecules could reduce the damage caused by this disinfectant. Finally, due to the reactivity rate of HOCl with proteins, it might affect membrane transport proteins, affecting the cell homeostasis; thus, suggesting that the downregulation of these proteins could be a protective strategy against the damage caused by HOCl. Figure 6 shows a schematic representation of the general mechanisms involved in biofilm stimulation by HOCl.

Together, these findings suggest that Gram-negative bacteria in biofilms share response mechanisms with planktonic cells and that many of these adaptative responses are not specific to HOCl but are also survival strategies against ROS. However, little is known about the regulatory systems employed by Gram-negative biofilms to overcome HOCl oxidative stress, and studies focusing on these mechanisms are still in their infancy, mainly due to the complexity of biofilm structures.

### 3.4. Sublethal Concentrations of HOCl Induce Antimicrobial Resistance and Virulence Gene Expression and VBNC

In addition to the adaptive responses involved in the elimination of HOCl presented above, several studies have shown that this chlorinating agent also induces cellular mechanisms associated with virulence and resistance. For instance, it has been demonstrated that chlorination induces antibiotic resistance in bacteria mainly by the overexpression of efflux pumps, stress resistance genes, such as *rpoS*, *marA*, *ygfA*, *relE*, and antibiotic resistance genes (ARGs) [174,175,176,177]. Hou et al. (2019) showed that previous exposure of *P. aeruginosa* cells to sublethal concentration (4 mg/L) of NaOCl increased the resistance of the bacteria to ceftazidime, chloramphenicol, and ampicillin (1.4–5.6 fold compared to the control) as a consequence of the upregulation of the efflux pump MexEF-OprN [174]. MexEF-OprN belongs to the resistance nodulation cells division (RDN) family and has been described to be involved in the resistance to fluoroquinolones, chloramphenicol, trimethoprim, and carbapenems [178]. The upregulation of genes involved in the expression of efflux pumps by HOCl has also been described in other studies [167,170]. For instance, Farrant and collaborators (2020) showed that MexEF-OprN mutants were more sensitive to the effects of HOCl than the wild-type [167]. In this context, multidrug efflux pumps could be involved not only in the export of antimicrobial agents and disinfectants but also in the transport of toxic HOCl sub-products generated by the reaction of this disinfectant with intracellular molecules.

In addition, Zhang et al. (2017) found that the use of NaOCl induces antibiotic resistance by increasing the rate of horizontal transfer of ARGs in *E. coli* and *S. typhimurium* [175]. The authors showed that this phenomenon is explained by the generation of intracellular ROS by NaOCl, which increases membrane permeability and alters the expression of genes involved in the conjugation process. For example, NaOCl decreases the expression of global regulatory genes, which promotes the conjugative transfer of plasmids [175]. These results indicate that gene transfer induced by HOCl exposure might be implicated in bacterial dissemination and resistance in several environments, including the host and drinking-water distribution and storage systems.

HOCl and NaOCl also induce the viable but nonculturable (VBNC) state in bacterial cells [176,179,180]. This condition is a bacterial response to hostile environments in which they enter in a dormant state where the cells remain viable but do not grow under standard culture conditions [181]. Microorganisms in this state possess many characteristics of viable cells, such as cellular integrity and activity, metabolic activity, and gene expression, and can recover once the stress condition is removed [182]. Moreover, VBNC cells can tolerate high doses of antibiotics, increased levels of pH, heat, ethanol, and heavy metals [181]. Several mechanisms could be associated with the induction of the VBNC state, namely: (i) overproduction of alarmone guanosine pentaphosphate [(p)ppGpp], which suppresses several cellular mechanisms that expend energy and resources; (ii) intracellular proteolysis; (iii) synthesis of toxin-antitoxin systems; (iv) decreased ATP levels; (v) cell deterioration; and (vi) genetic regulation (for more details see [181]) [181,183,184]. Regarding the genetic control of the VBNC state, RpoS, a regulator involved in the bacterial survival under different environmental conditions, has been described to be involved in the formation of the VBNC state [183]. Lin et al. (2017) showed that 0.5 mg/L of NaOCl induced the VBNC state of *E. coli* after 6 h and that VBNC *E. coli* was more tolerant to antibiotics than culturable bacteria. The authors showed that in VBNC bacteria, the levels of transcription of *rpoS* was 3.48-fold higher. In addition, other genes involved in stress resistance (e.g., *marA*, *ygfA*, and *relE*), ARGs, porin, and efflux pumps were also upregulated in VBNC bacteria, indicating a low metabolic activity and the presence of efflux genes could be involved in the high tolerance of VBNC cells to antibiotics [176]. In general, the presence of the VBNC state has several implications. For example, the presence of VBNC can hinder bacterial detection as well as cause severe infections if they return to the active state [185].

In addition to the stimulation of bacterial resistance, HOCl also induces cellular responses involved in virulence. For instance, Huang et al. (2014) showed that the concentrations of virulence proteins, such as flagellar motor switch protein (FliG), Clp protease, and inner membrane protein OxaA, in water after chlorination increased by 1.7 fold compared to filtered water due to the horizontal gene transfer among the surviving bacteria [186].

Furthermore, several studies have described cellular responses by analyzing the bacterial genome-wide transcription changes upon exposure to HOCl. In this context, exposure of *P. aeruginosa* cells to HOCl marked downregulated genes involved in energy production, including those involved in oxidative phosphorylation and the electron transport chain, suggesting that cells exposed to HOCl decrease their energy production. On the other hand, genes involved in the transport of sulfur, membrane proteins, and putative enzymes were considerably upregulated [75,80]. Farrant et al. (2020) found that the most upregulated genes in *P. aeruginosa* in response to HOCl exposure were non-coding RNA genes, followed by genes associated with antibiotic resistance, and protein secretion and export systems. Among the genes involved in the transport of molecules, the highly overexpressed were those involved in sulfur and taurine metabolism, indicating that bacterial cells use taurine as a sulfur source under sulfur starvation conditions, thereby the uptake of those substances should increase [167]. Additionally, exposure to HOCl also activates virulence systems used to overcome the host immune system. In this context, the induction of pyocyanin production by *P. aeruginosa* and the activation of the type 3 secretion system (T3SS) were reported to be an adaptive response to HOCl-induced stress [167] since they are involved in necrosis and apoptosis of immune cells (*i.e.*, neutrophils and macrophages) [187,188]. T3SS is an important virulence factor widely found in Gram-negative bacteria. It consists of rings that provide a continuous path across the bacterial inner and outer membranes and the host cell membrane, which enables bacteria to inject effectors into the host cell. The T3SS and its effectors are involved in several cellular processes, including host invasion, host immune responses, and vesicle transport [188,189].

## 4. Final Remarks

Although bacteria are resistant to several classes of antimicrobial agents, they are constantly confronted by oxidizing agents that can cause irreversible damage to the cells. Therefore, in order to respond to the damage caused by these agents, especially HOCl, bacteria undergo several cellular adjustments, including the activation of transcriptional regulators and chaperones. This adaptive response is a complex regulatory network in which several genes with a variety of functions are suppressed or induced. However, although the knowledge of how bacteria sense and respond to HOCl has gradually increased over time, these response mechanisms have not been completely elucidated, and the majority of the studies focus on ROS stress response. Furthermore, several transcriptional sensors that are involved in ROS resistance have also been described to be involved in HOCl-induced response. In this context, only three HOCl-specific regulators (HypT, NemR, and RclR) have been identified in Gram-negative bacteria so far. The affinity of these regulators to HOCl can be explained by the high reaction rate of this disinfectant with thiol groups in comparison to H_2_O_2_.

Additionally, due to the quick oxidation of proteins and the formation of unfolded molecules and ATP depletion, the activation of chaperone holdases is considered an immediate response against the proteome damage caused by HOCl. Another crucial adaptive response to HOCl is the formation of biofilms. Studies have shown that sub-lethal concentrations of HOCl induce the formation of robust biofilms. However, the mechanisms involved in this process have been poorly explored. In addition, the acquisition of antibiotic resistance by cells exposed to HOCl and its effect on virulence systems have proved that this chlorinating agent is involved in complex and diverse adaptive responses and have several health implications. Therefore, these stress responses adopted by cells against HOCl disinfection highlights the need for effective strategies to control bacterial colonization after disinfection in the water treatment systems, industry, and health facilities. For instance, the adoption of standard hygiene protocols that focus on the safe use of disinfectants, such as the concentration and exposure time, especially in health care facilities where poor hygiene might enhance bacterial spread, could be an effective way to control bacterial colonization.
